# Etymologia: ***Clostridium difficile***

**DOI:** 10.3201/eid1604.ET1604

**Published:** 2010-04

**Keywords:** etymology, Etymologia, *c. difficle*, *clostridium difficile*

##  [klos-trid′e-əm di-fi -sil′]

*Clostridium*, the genus name of these gram-positive, spore-forming, anaerobic bacteria comes from Greek *klōstēr* (spindle) because, under the microscope, the colonies resemble spindles used in cloth weaving and long sticks with a bulge at the end. The species name *difficile* is a form of the Latin adjective *difficilis* because when first identified (by Hall and O’Toole in 1935), the organism was difficult to isolate and grew slowly in pure culture. However, likely because of the familiarity of a French term with the same spelling and meaning, the French pronunciation has become widely used. These bacteria are part of the commensal intestinal flora in humans, and toxigenic strains of the organism can cause pseudomembranous colitis, a severe infection of the colon, after normal gut flora have been eradicated in patients who have received antimicrobial drugs.

Sources: Kelly CP, Pothoulakis C, LaMont JT; *Clostridium difficile* colitis. N Engl J Med. 1994;330:257–62; Wells J. My phonetic blog. 2006. www.phon.ucl.ac.uk/home/wells/blog0606.htm; www.statemaster.com/encyclopedia/Clostridium-difficile; Dorland’s illustrated medical dictionary, 31st ed. Philadelphia: Saunders Elsevier; 2007.

